# Subchronic Hepatotoxicity Evaluation of 2,3,4,6-Tetrachlorophenol in Sprague Dawley Rats

**DOI:** 10.1155/2012/376246

**Published:** 2012-05-15

**Authors:** Darol E. Dodd, Linda J. Pluta, Mark A. Sochaski, Deborah A. Banas, Russell S. Thomas

**Affiliations:** ^1^Institute for Chemical Safety Sciences, The Hamner Institutes for Health Sciences, Research Triangle Park, NC 27709-2137, USA; ^2^Virginia Pathology, Experimental Pathology Laboratories, Inc., Sterling, VA 20167-0169, USA

## Abstract

Male Sprague Dawley rats were exposed to 2,3,4,6-tetrachlorophenol (TCP) for 5 days, 2 weeks, 4 weeks, or 13 weeks. TCP was administered by gavage at doses of 0, 10, 25, 50, 100, or 200 mg/kg/day. Endpoints evaluated included clinical observations, body weights, liver weights, serum chemistry, blood TCP, gross pathology, and liver histopathology. There were no TCP exposure-related clinical signs of toxicity. Mean body weight decreased 12–22% compared to control in the 100 and 200 mg/kg/day groups. Serum ALT concentrations were increased in rats of the 200 mg/k/day. Liver weight increases were both dose- and exposure time-related and statistically significant at ≥25 mg/kg/day. Incidence and severity of centrilobular hepatocytic vacuolation, hepatocyte hypertrophy, and single cell hepatocytic necrosis were related to dose and exposure time. Following 13 weeks of exposure, bile duct hyperplasia and centrilobular and/or periportal fibrosis were observed in rats primarily of the highest TCP dose group. Blood TCP concentrations increased with dose and at 13 weeks ranged from 1.3 to 8.5 **μ**g/mL (10 to 200 mg/kg/day). A NOAEL of 10 mg/kg/day was selected based on the statistically significant incidence of hepatocyte hypertrophy at doses ≥25 mg/kg/day.

## 1. Introduction

Chlorophenols are toxic to a wide range of organisms and, therefore, are manufactured primarily to be used as bactericides, insecticides, or fungicides in a variety of commercial products. For example, mixtures of pentachlorophenols and tetrachlorophenols, including the chemical 2,3,4,6-tetrachlorophenol (TCP) (CAS number 58-90-2), are used at sawmills as wood preservatives [1–3]. Routes of potential human exposure of chlorophenols are dermal contact, inhalation, and ingestion. Urinary concentrations of pentachlorophenol (PCP) and TCP have been observed in woodworkers exposed to wood preservative mixtures of PCP and TCP [4]. Nonoccupationally exposed humans in regions of Finland had trace amounts of TCP in adipose and liver tissues [5]. TCP has a relatively high octanol-to-water partition coefficient causing a concern for bioaccumulation. Soil and downstream water samples from contaminated sawmill sites contain chlorophenols, including TCP [3, 6]. Chlorophenols have been detected in chlorinated drinking water and city air. The U.S. Environmental Protection Agency (EPA) has issued recommendations for drinking water concentrations of several chlorophenols. The recommendation for TCP in drinking water is 1 ppb, though extensive investigation of the health effects of TCP has not been performed. A thorough summary of health effects of chlorophenols, including TCP, in humans and laboratory animals was published by the Agency for Toxic Substances and Disease Registry (ATSDR) in 1999 [7].

Acute and subchronic oral toxicity studies in rats using TCP were conducted by Hattula et al. [8]. Acute gavage doses ranged from 300 to 632 mg/kg (TCP dissolved in olive oil), and subchronic (7 days/week for 55 days) doses ranged from 10 to 100 mg/kg/day. Multiple tissues were collected at necropsy and examined microscopically. Results indicated that the most adverse histopathological changes were observed in the liver following either single or repeated doses of TCP. Liver effects were dose dependent and ranged from mild (mild proliferation of the bile canaliculi and occasional necrosis of cells of the canaliculi and hepatocytes) to medium (focal areas of necrosis, large basophilic hepatocytes, and gathering of polymorphonuclear leukocytes) to severe (large confluent necrosis covering most of the liver parenchyma and dilated veins) in severity. Inflammation of hepatic bile ducts was also observed. EPA conducted a 90-day oral toxicity study in Sprague Dawley (SD) rats using TCP doses of 0, 25, 100, or 200 mg/kg/day [9]. Liver weights were increased and centrilobular hypertrophy was observed in rats of the two highest TCP doses. A NOAEL of 25 mg/kg/day was selected.

Developmental toxicity studies in SD rats indicated that TCP (purified or commercial grade) was fetotoxic (delayed ossification of the skull bones) at an oral dose of 30 mg/kg/day, but not teratogenic [10]. A NOAEL for embryotoxicity was 10 mg/kg/day. A second developmental toxicity study in SD rats was conducted by EPA and used oral TCP doses of 0, 25, 100, or 200 mg/kg/day [11]. Maternal weight gain was reduced at 200 mg/kg/day, but embryofetal growth, prenatal viability, and fetal development were not adversely affected. Genotoxicity results of TCP were mixed showing both positive and negative findings in several *in vitro* assays [7]. Oxidative stress [12] and the absence or presence of microsomal activation enzymes [13] were likely contributing factors to uncertainty of a direct mutagenic effect. Based on results of Kitchin and Brown [14] who administered acute oral doses of TCP in rats, TCP may be a weak promoter. Ahlborg and Larsson [15] investigated the metabolism of three tetrachlorophenols (including the 2,3,4,6-isomer) in rats following intraperitoneal administration and identified hydroquinone metabolites in the urine. The majority of the TCP dose (96%) was excreted unchanged in the urine [15]. TCP is a metabolite of PCP and chlorobenzenes, such as pentachlorobenzene [16, 17].

Currently, TCP is listed in the Integrated Risk Information System (IRIS) database with a chronic oral reference dose (RfD) of 0.03 mg/kg/day [18]. The critical noncarcinogenic effects used for the point of departure in calculating the RfD were increased liver weights and centrilobular hypertrophy. The objective of this study was to focus on liver toxicity associated with oral TCP exposure and characterize both the dose response and time course relationships. The choice of liver toxicity was due to its selection as the critical effect in the noncancer IRIS risk assessment. Previous studies with TCP had not fully characterized the temporal relationship of the progression of liver effects especially at TCP doses that included and extended beyond (both below and above) previously reported NOAEL and LOAEL doses. Five doses of TCP and 4 time points were selected to assess liver toxicity in male Sprague Dawley rats. Endpoints evaluated included clinical observations, body weights, liver weights, serum chemistry, gross pathology, and liver histopathology. In addition, blood samples collected at necropsy were analyzed for TCP. Information gained from this study will be used to inform selection of the critical adverse effect of TCP, evaluate its progression, and select a NOAEL.

## 2. Materials and Methods

### 2.1. Test Materials

2,3,4,6-tetrachlorophenol (CAS No. 58-90-2), 98% purity, was obtained from International Laboratories USA (Catalog No. 1618611). Olive oil (CAS No. 8001-25-0) was obtained from VWR Scientific (Radnor, PA; Catalog number 95034-796). Both test materials were stored at room temperature until use.

2,3,4,6-tetrachlorophenol (TCP) purity was verified by analysis of the neat compound dissolved in hexane using gas chromatography with flame ionization detection (GC/FID). Compound purity was calculated to be >99.9% TCP. Qualitative analysis of the parent compound was conducted by GC/mass spectrometry (MS) (Agilent 6980 GC coupled to an Agilent 5973 MS) in an attempt to identify any impurities present. Observed impurities included multiple tetrachlorophenol isomers (minor), trichlorophenol (minor), and 3-4 chlorinated compounds which were not identified by the mass spectral library database (trace).

### 2.2. Study Design, Animals, and Animal Husbandry

The Hamner Institutes for Health Sciences is fully accredited by the Association for Assessment and Accreditation of Laboratory Animal Care International (AAALAC). Currently acceptable practices of good animal husbandry were followed per National Research Council's *Guide for the Care and Use of Laboratory Animals *[19] and were in compliance with all appropriate parts of the Animal Welfare Act regulations [20, 21]. In addition, the study design and protocol were approved by The Hamner Institutes' Institutional Animal Care and Use Committee (IACUC) prior to the initiation of study.

Two hundred and forty-five 4-5-week-old male Sprague Dawley rats (Rat/Crl:CD(SD)) from Charles River Laboratories (Raleigh, NC) were used in this study (Table 1). An additional one or two rats were assigned to the control group per time point to be used to assure a group size of at least 10 for evaluation of biological endpoints. Upon arrival, rats were acclimated to housing and animal room environment for 12–14 days. Rats were weighed and randomized using Provantis 8 (Provantis, Conshohocken, PA) to ensure that mean body weight in each treatment group was approximately the same. Animals were ear-tagged and housed 2 or less rats per cage in shoebox style cages separated by treatment group. Alpha-dri cellulose bedding (Shepard Specialty Papers, Kalamazoo, MI) was used. Animals had access to reverse osmosis water (Hydro Systems, Durham, NC) and pellet NIH-07 certified feed (Zeigler Brothers, Gardners, PA) *ad libitum*. The animal room was kept within the standard temperature and relative humidity parameters (64 to 79°F and 30 to 70% relative humidity) and standard light cycle (0700–1900 hours).

### 2.3. Preparation and Administration of TCP

Doses of TCP selected for study were 0, 10, 25, 50, 100, and 200 mg/kg/day. Dose selections were similar to those used in EPA's 90-day oral gavage study in Sprague-Dawley rats [9] of 0 to 200 mg/kg/day but extended lower (10 mg/kg/day) to assure a NOAEL dose group and to be in agreement with IRIS [18] point of departure dose level of 25 mg/kg/day for deriving an oral reference dose (RfD). Dose selections did not exceed 200 mg/kg/day, since TCP-exposure-related depression of body weights was observed at 200 mg/kg/day in the EPA study [9].

Dosing solutions of TCP were prepared in olive oil and administered by oral gavage at a volume of 5 mL/kg body weight. Dosing solutions were prepared weekly (approximately) and submitted for analysis on the same day they were prepared. Upon receipt, dosing solutions were diluted in triplicate with a sufficient amount of nanograde hexane (Mallinckrodt, St.Louis, MO) to allow the theoretical dose concentrations to fall within a freshly prepared calibration curve. The TCP calibration curve was prepared in olive oil and diluted to a final concentration range of 45 to 270 *μ*g/mL with hexane. Samples and standards were analyzed by GC/FID using a 60 m × 320 *μ*m × 0.25 *μ*m Restek Rtx-1 column (Restek, Bellefonte, PA) and helium as the carrier gas. Analysis was carried out in split mode (3 : 1) with a total flow rate of 11.0 mL/min and an injector temperature of 250°C. The GC temperature gradient used was as follows: (1) initial temperature was held at 150°C for 1 min; (2) following the 1 min hold, the temperature was ramped up at 25°C/min until the final temperature of 300°C was obtained; (3) the final temperature was held for 4 min for a total analysis time of 11 min. Chromatograms were manually integrated and dosing solution concentrations were calculated through the use of the generated calibration curves and dilution factors.

Dosing solution stability for TCP was conducted at two concentrations, 2 and 40 mg/mL in olive oil, and under three different environmental conditions. Aliquots of the prepared doses were stored at (1) room temperature, (2) room temperature and covered in aluminum foil, or (3) 4°C and covered in aluminum foil. Sub-samples of the aliquots were analyzed by GC/FID following dilution with hexane (final concentration of 100 and 200 *μ*g/mL after dilution) and quantified through comparison to a freshly prepared calibration curve (described previously). Sample analysis was conducted every week for a total of 4 weeks (4 total analyses including time 0). Following completion of the stability studies, it was observed that the concentration of TCP in olive oil stored under cold storage conditions decreased slightly more than the TCP concentrations stored under the other two conditions. However, all three storage conditions were still within ±10% of the original time 0 concentration.

The mean ± standard deviation of the weekly dosing solutions was 2.0 ± 0.1, 4.7 ± 0.8, 9.7 ± 1.3, 19.5 ± 3.6, and 39.1 ± 2.4 mg TCP/mL corn oil for target concentrations of 2, 5, 10, 20, and 40 mg TCP/mL olive oil, respectively. Animals were orally gavaged with 5 mL of dosing solution/kg body weight resulting in weekly mean analytical doses of 10.0, 23.5, 48.5, 97.5, and 195.5 mg TCP/kg body weight. Control animals received 5 mL/kg body weight olive oil vehicle only. Gavage exposure occurred in the morning, 7 days/week, for the selected time period (Table 1).

### 2.4. Mortality Checks, Clinical Observations, Body Weights, and Organ Weights

Animals were checked daily for clinical signs of toxicity, morbidity, or death. Body weights were measured daily just before gavage dosing and prior to scheduled necropsy. At necropsy, the liver was removed and weighed. Clinical observations, body weights, and organ weights were recorded in Provantis 8.

### 2.5. Necropsy, Serum Clinical Chemistry, Blood TCP Analysis, and Tissue Histopathology

Animal necropsies occurred on scheduled days (Table 1) within a few hours following the administration of TCP (or olive oil for controls). Animals were weighed and anesthetized with a lethal intraperitoneal injection of sodium pentobarbital. A cardiac puncture was performed to collect blood samples, and the animal was then exsanguinated via transection of the abdominal aorta. Blood samples were placed in a serum separator tube (gel barrier) and centrifuged for clinical chemistry analysis. The following analytes were measured in the serum within a few hours of collection: aspartate aminotransferase (AST), alanine aminotransferase (ALT), alkaline phosphatase (ALP), bilirubin (total), and lactate dehydrogenase (LDH). Reagent sets for the serum analytes and quality control (QC) materials, including standards were obtained from Pointe Scientific, Inc. (Canton, MI). Instructions supplied with the reagent sets were followed. A Roche COBAS FARA II chemistry analyzer was used for analysis of serum samples. The COBAS operator manual and laboratory standard operating procedures (SOPs) were followed for conducting analyte analyses.

Another aliquot of at least 0.5 mL of blood was collected from at least 5 animals per dose per time point (animals selected randomly), placed in an anticoagulated tube (EDTA), and stored in a freezer (−80°C). These samples were analyzed for TCP concentration using GC with electron capture detection (GC/ECD). Aliquots (250 *μ*L) of thawed whole blood samples were mixed with 250 *μ*L of saturated sodium chloride solution and 10 *μ*L of internal standard solution (Lindane in methanol at 25 *μ*g/mL). Samples were then extracted with 500 *μ*L of hexane, briefly vortexed, placed on a sample rotator for 30 min at room temperature, and allowed to sit for 5 min prior to centrifugation at 8000 × G for 5 min. The top hexane layer was removed and transferred to a GC sample vial for analysis. Calibration curve samples were prepared by spiking whole blood with known amounts of TCP to a range of 1.9 to 24 *μ*g/mL. The limit of quantitation for TCP was below 0.3 *μ*g/mL blood. Samples and standards were analyzed on a 30 m × 320 *μ*m × 0.25 *μ*m Restek Rtx-1 column using helium as the carrier gas. Instrumental analysis was carried out in splitless mode with a total flow rate of 8.5 mL/min and an injector temperature of 250°C. The GC temperature gradient used was as follows: (1) initial temperature was held at 150°C for 1 min; (2) following the 1 min hold, the temperature was ramped up at 25°C/min until the final temperature of 300°C was obtained; (3) the final temperature was held for 4 min for a total analysis time of 11 min. Chromatograms were manually integrated and dosing solution concentrations were calculated through the use of the generated calibration curves and dilution factors.

Following gross examination for abnormalities, a slice from 3 of the 4 liver lobes (median, right, and left) was placed in a labeled cassette. The liver lobes were not evaluated independently to identify or assess interlobe variability but were evaluated together to characterize an overall hepatic effect of TCP exposure. The cassette was placed into a 10% neutral buffered formalin cup for approximately 48 hours. The cassette was then transferred to a cup containing 70% ethanol followed by paraffin embedding. Liver-embedded cross sections (5 um) were stained with hematoxylin and eosin for microscopic evaluation by a board-certified pathologist. Histopathology observations were ranked based on the following severity score: 1, minimal; 2, slight/mild; 3, moderate; 4, moderately severe; and 5, severe/high. An average severity score was calculated by totaling the severity scores for an observation at a specified exposure site for an exposure cohort and dividing by the total number of animals affected.

### 2.6. Statistical Analysis

Body weight and organ weight data were analyzed using the statistical module of the Provantis software data collection system (NT2000 versions 8.2.0.1 or 8.2.0.6, Instem, Coshohoken, PA). A one-way analysis of variance (ANOVA) test followed by a Dunnett's test was used to compare the control group with TCP exposure groups at each time point. Serum chemistry data were analyzed using JMP 9.0.0 software (SAS Institute, Inc., Cary, NC). A goodness of fit test (Shapiro-Wilk, *P* < 0.01) and homogeneity of variances test (Levene's, *P* < 0.05) were conducted. If pretest assumptions were met, an ANOVA was used and, if significant (*P* < 0.05), TCP-exposed groups were compared to the control group using Dunnett's test. For data sets of nonnormal distributions or unequal variances, a Welch ANOVA followed by Steel's test was used. For incidence data (histopathology), a Fisher's exact test (one tail) was done comparing each TCP dose group to the corresponding control group and applying the Bonferroni correction to *P* values. A result of *P* < 0.05 was considered significant.

## 3. Results

### 3.1. Clinical Observations and Body Weights

There were no TCP exposure-related clinical signs of toxicity during the study. Statistically significant decreases were observed in mean body weights of rats of the 200 mg/kg/day group compared to controls beginning week 7 of dosing. By study week 13, the mean body weight was 22% lower than control animals. Mean body weights were also reduced in animals of the 100 mg/kg/day group compared to control and were statistically significant during the last 3 weeks of dosing (study weeks 11, 12, and 13). By study week 13, the mean body weight was 12% lower in the 100 mg/kg/day group compared to control animals. Mean body weights of rats of the 50 mg/kg/day group were mildly reduced (6%) compared to controls during the study, but these reductions were not statistically significant. Mean body weights were similar between control and TCP exposure groups of ≤25 mg/kg/day throughout the study. Individual and group mean body weights for all animals on study at each scheduled necropsy are provided in Supplemental Table 1 in Supplementary Material available online at doi:10.1155/2012/376246. 

### 3.2. TCP in Blood

Blood samples for TCP analysis were collected approximately two hours after dosing at each time point. Blood TCP concentrations increased with dose. For example, at 13 weeks, mean TCP blood concentrations were 1.3, 2.2, 5.3, 8.7, and 8.5 *μ*g/mL for TCP dose groups of 10, 25, 50, 100, and 200 mg/kg/day, respectively (Table 2). Mean TCP blood concentrations were lower after 4 or 13 weeks exposure, compared to TCP blood concentrations following 5 days or 2 weeks exposure (Table 2).

### 3.3. Serum Chemistry

Statistically significant increases in mean ALT in the 200 mg/kg/day group compared to control values were observed following 2, 4, and 13 weeks of exposure (Table 3). At 13 weeks, ALT was also increased in the 50 and 100 mg/kg/day exposure groups. Increases in mean ALP and AST were also observed in the 200 mg/kg/day group at 13 weeks (*P* < 0.01), but not at other exposure time points or in lower TCP dose groups (≤100 mg/kg/day) (Table 3). There were no TCP exposure-related increases in LDH and total bilirubin compared to control values throughout the study (data not shown).

### 3.4. Organ Weights

At TCP doses ≥100 mg/kg/day, statistically significant increases in absolute and relative (to body weight) mean liver weights compared to controls were observed in rats at all time points (Table 4). As early as 5 day exposure, statistically significant increases in absolute and relative mean liver weights were observed in the 100 and 200 mg/kg/day groups. Significant increases in mean liver weights were observed in the 50 mg/kg/day group following 2-, 4-, or 13-weeks exposure. Mean liver weights were significantly increased in rats of the 25 mg/kg/day group following 2 and 13 weeks of TCP exposure. At the 2-week necropsy, relative liver weights were increased (*P* < 0.05) compared to controls in rats of the 10 mg/kg/day group, but at no other time point (Table 4). The liver weight increases were both TCP dose dependent and exposure time dependent (Table 4). For example, at the 4-week necropsy, the mean relative liver weights, expressed as percentage of control, were 99%, 108%, 121%, 147%, and 168% for the 10, 25, 50, 100, and 200 mg/kg/day groups, respectively. At 13 weeks, percentage of control values was 118%, 141%, 176%, 229%, and 303% for the 10, 25, 50, 100, and 200 mg/kg/day groups, respectively. Individual animal liver weight data are provided in Supplementalary Table 1.

### 3.5. Histopathology

At the scheduled necropsies, there were isolated gross lesions observed in livers of a few TCP-exposed animals that consisted of areas of pale appearance, dark appearance, or at the 13-week sacrifice, a uniform brown parenchyma. The pale appearance was noted in a few rats and corresponded to scattered vacuolation. The dark appearance was due to passive congestion in the liver and was not diagnosed. Brown parenchyma of the liver was present in 80% of the rats of the 200 mg/kg/day group at the 13-week necropsy. This finding corresponded to moderately severe to severe scoring of centrilobular hepatocytic vacuolation. The remaining animals in the 200 mg/kg/day group that did not have the gross appearance of brown parenchyma also had centrilobular hepatocytic vacuolation, but the score was less severe.

**Figure 1:**
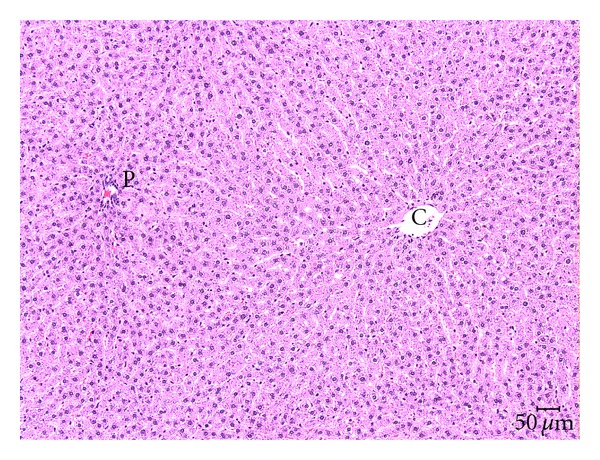
Normal liver (0 mg/kg/day TCP following 13 weeks exposure) showing uniform size and shape of hepatocytes around the central vein (C) and portal area (P). H & E, 10x.

**Figure 2:**
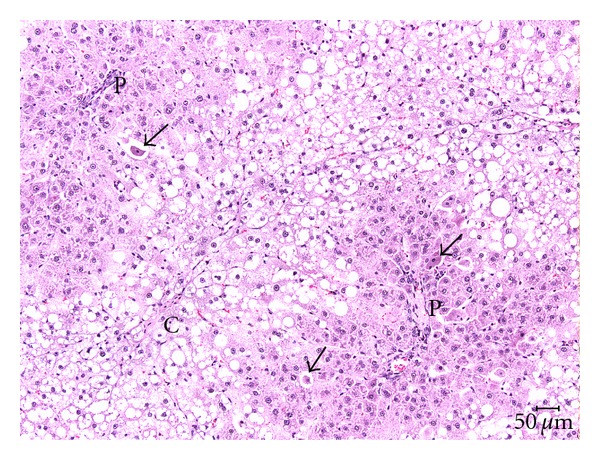
Liver (200 mg/kg/day TCP following 13 weeks exposure) characterized by marked hepatocellular hypertrophy and hepatocellular vacuolation bridging from one centrilobular area (C) to the next. Both the portal (P) and centrilobular veins (C) are compressed. Single cell necrosis is indicated by arrows. H & E, 10x.

**Figure 3:**
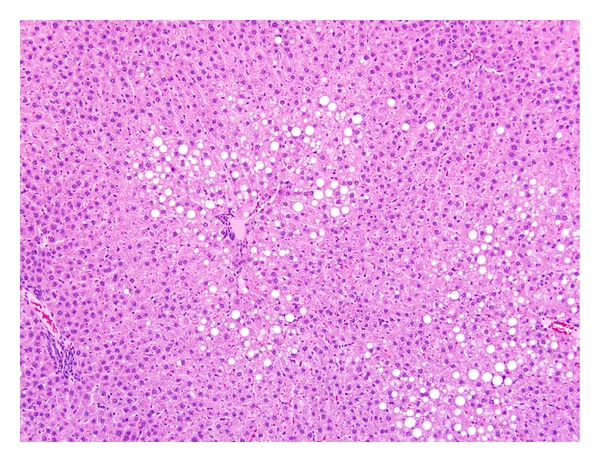
Liver (25 mg/kg/day TCP following 13 weeks of exposure) characterized by mild hepatocellular hypertrophy and hepatocellular vacuolation around the central veins. H & E, 10x.

TCP-related liver microscopic alterations that were observed as early as 5 days exposure and gradually became higher in incidence and more severe in appearance were centrilobular hepatocytic hypertrophy and single cell hepatocytic necrosis (Table 5). In the two highest doses of TCP, the hypertrophy was diffuse and the single cell necrosis was midzonal following 13 weeks of exposure (Figures 1 and 2). Centrilobular single cell necrosis and hepatocytic hypertrophy were also observed in animals of the 50 mg/kg/day dose group after 2 weeks of exposure, but with less incidence (20–40%) and severity (minimal) compared to the two higher TCP dose groups. Single cell necrosis was not observed in rats of ≤25 mg/kg/day, but hepatocytic hypertrophy was observed in rats of the 25 mg/kg/day group following 4 weeks or 13 weeks of TCP exposure (Figure 3 and Table 5). Four rats of the 10 mg/kg/day group had minimal hepatocytic hypertrophy, but the incidence was not statistically significantly different compared to rats of the control group.

**Figure 4:**
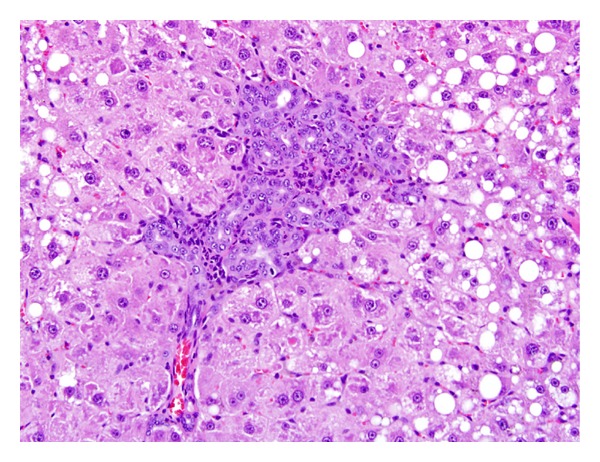
Liver (200 mg/kg/day following 13 weeks of exposure) characterized by diffuse hepatocellular hypertrophy, hepatocellular vacuolation, and biliary ductal hyperplasia in the portal area. H & E, 20X.

**Figure 5:**
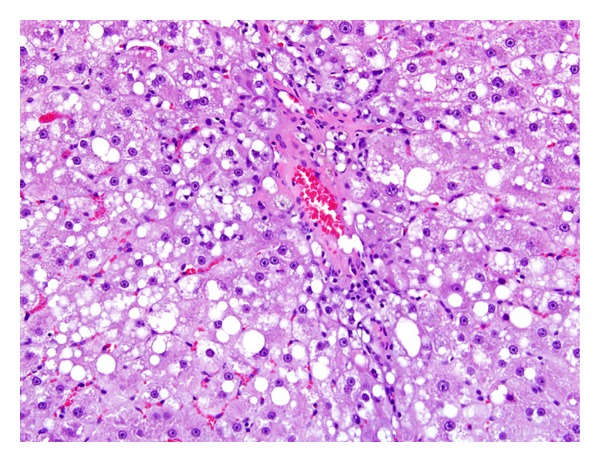
Liver (200 mg/kg/day following 13 weeks of exposure) characterized by hepatocellular hypertrophy and hepatocellular vacuolation, with vacuoles of varying sizes, around a central vein which has early fibrosis. H & E, 20x.

**Figure 6:**
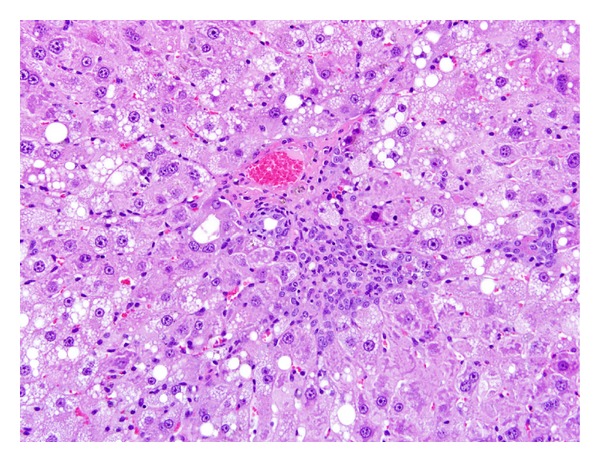
Liver (200 mg/kg/day following 13 weeks of exposure) characterized by hepatocellular hypertrophy and vacuolation, biliary ductal hyperplasia, and periportal fibrosis. H & E, 20x.

As early as 2 weeks of exposure, centrilobular hepatocytic vacuolation was a consistent finding in TCP-exposed rats of ≥25 mg/kg/day. However, scattered or centrilobular hepatocytic vacuolation was also observed in control rats at the 4-week and 13-week necropsies. The incidence and severity of vacuolation were greater in TCP exposure groups indicating exacerbation of this alteration with time and dose. Hepatocytic vacuolation was the most prominent finding in TCP-exposed rats at the 13-week necropsy. In rats of the 200 mg/kg/day group, hepatocytic vacuolation extended well into the midzonal areas and formed bridges from one centrilobular area to another (Figures 1 and 2). Less severe hepatocytic vacuolation was noted at 25 mg/kg/day (Figure 3). Bile duct hyperplasia was observed with 100% incidence in rats of the 200 mg/kg/day group at 13 weeks; incidence was 20% in rats of the 100 and 25 mg/kg/day groups (Figure 4). This change appeared to be a secondary response to injury to the hepatocytes. Changes in the bile canaliculi or sinusoids were not noted in any of the animals examined. Incidences of 40 to 60% centrilobular and/or periportal fibrosis were observed in rats of the 200 mg/kg/day group, and 10% incidence was observed in rats of the 100 and 25 mg/kg/day groups (Figures 5 and 6).

## 4. Discussion

In previous rodent oral gavage toxicity studies with TCP, the liver was identified as the critical target organ of effect following acute or repeated exposure. Increases in liver weights, serum ALP, ALT, bilirubin, and hepatic inflammation, hypertrophy, and necrosis were reported [8, 9]. The results obtained in the current study are in agreement with these studies and provide noteworthy new information about dose and exposure duration related to TCP toxicity. For example, body weight reduction and increased liver weights were observed in rats of the highest dose groups (100 and 200 mg/kg/day) in the current 13-week study and the EPA 90-day study [9]. In the current study, increased liver weights were also observed in rats of lower TCP dose groups (50 and 25 mg/kg/day) following 13 weeks of exposure, though this result was not observed in rats dosed with 25 mg TCP/kg/day in the EPA study [9]. Serum ALT levels were elevated in male rats of the 200 mg/kg/day group following 13 weeks exposure (current study and EPA study [9]), but the current study also showed an increase in serum ALT after 2 or 4 weeks of exposure at this dose and an increase in ALT in rats of the 50 and 100 mg/kg/day groups after 13 weeks of exposure. Serum ALP and AST were significantly increased in rats of the high-dose group at 13 weeks (Table 3), but this was not completely consistent with findings in the EPA study [9], where ALP increased in male rats of the 200 mg/kg/day group after 45 days of exposure only. Also, serum total protein and albumin were increased in male rats of the 100 and 200 mg/kg/day groups after 90 days of exposure [9]. Results of both current and EPA studies indicate that at high doses of TCP (≥50 mg/kg/day) serum markers of liver injury were increased with ALT being the most consistent marker of effect. The lack of hepatocyte necrosis (Table 5, TCP doses ≤ 25 mg/kg/day, all time points) or mild degree of necrosis (Table 5, TCP doses of 50 and 100 mg/kg/day, all time points) may explain the absence or weak response of the serum enzyme findings (Table 3). Serum ALP, ALT, and AST were significantly increased at 200 mg/kg/day following 13 weeks of exposure, and this observation correlated well with the incidence (100%), degree (mild/moderate), and extent (midzonal) of hepatocyte necrosis.

The most notable difference in results between the current study and previous studies is the observation of liver histopathology at lower TCP doses (≤25 mg/kg/day). Centrilobular hypertrophy and vacuolation were observed in rats of the 10 and 25 mg/kg/day groups (Table 5). However, after 13 weeks of exposure, the severity of hypertrophy was minimal in the 10 mg/kg/day rats, and the incidence was not statistically significantly different compared to the control rats. Hepatocyte hypertrophy is typically observed with enzyme induction; although Phornchirasilp et al. [22] observed significant increases in microsomal protein and cytochrome P450 levels in Sprague-Dawley rats dosed with 4-chlorophenol, studies reporting TCP-induction of liver metabolizing enzymes were not found suggesting that other pathways may be involved in the hypertrophy response. The observed hepatocyte vacuolation in conjunction with hypertrophy (Table 5) may indicate perturbation of lipid metabolism, a common sequel to hepatic injury. Although specific lipid staining was not done in the present study, the appearance and distribution of the vacuoles in the liver are histologically consistent with lipid accumulation in injured hepatocytes. Lipid accumulation could have a number of causes although the most probable one is oxidative stress and biotransformation of TCP to electrophilic intermediates. Ahlborg and Larsson [15] administered Sprague-Dawley rats with TCP (i.p.) and identified trichloro-p-hydroquinone as a minor urinary metabolite. Further, Arrhenius et al. [23] reported disruption of microsomal detoxification enzymes by TCP, which may contribute to the initiation of free radicals and lipid peroxidation in the liver. The most obvious toxic mechanism associated with exposure of TCP and related chlorophenols (e.g., pentachlorophenol) is the uncoupling of mitochondrial oxidative phosphorylation [24]. The higher substituted chlorophenols, such as TCP, are more potent in uncoupling oxidative phosphorylation than the monochlorophenols [25, 26]. Drugs or chemicals that uncouple oxidative phosphorylation can produce liver hypertrophy [27].

A second notable difference in the histology of prior studies compared to this one was that alterations in bile canaliculi and diffuse necrosis of the liver described by Hattula et al. [8] were not evident in the animals examined on this study. Biliary changes were limited to bile duct hyperplasia in the portal area. This change appeared to be a secondary response due to injury to the hepatocytes. This apparent difference in toxic hepatic response in the prior study may be due to differences in rat strains or other experimental parameters.

With respect to the time course of liver weights, serum enzymes, and liver histopathology, the current study clearly showed a progression of greater severity of effect with increase in exposure duration of TCP (Tables 3, 4, and 5). For example, statistically significant increases of centrilobular hepatocytic hypertrophy were observed at 200 mg/kg/day after 5 days of exposure, at ≥100 mg/kg/day after 2 weeks of exposure, at ≥50 mg/kg/day after 4 weeks of exposure, and at ≥25 mg/kg/day after 13 weeks of exposure (Table 5). Severity also increased with time and by 13 weeks the hypertrophy was diffused in the 200 and 100 mg/kg/day groups (Table 5). Centrilobular single cell necrosis gradually became midzonal with time in the two highest dose groups. At 13 weeks, bile duct hyperplasia and centrilobular/periportal fibrosis were observed primarily in rats of the 200 mg/kg/day group (Figures 4, 5, and 6). Hattula et al. [8] observed proliferation of the bile canaliculi in rats dosed with TCP and attributed this alteration as a secondary process preceded by specific parenchymal cell damage. Centrilobular vacuolation was first observed in rats of the mid- and higher-dose groups (≥25 mg/kg/day) after 2 weeks of exposure and became prevalent in all TCP dose groups after 4 weeks of exposure. However, at 13 weeks, minimal vacuolation was noted in 33% of the controls. The olive oil vehicle received by the control rats may be a contributing factor to this observation. In the highest TCP dose groups, extensive bridging vacuolation resulting in brown color to the liver parenchyma was noted in rats during the 13-week necropsy.

In conclusion, male SD rats administered TCP by gavage at doses up to 200 mg/kg/day for up to 13 weeks had mild liver effects manifested as increased liver weights, centrilobular hepatocytic single cell necrosis, hepatocyte hypertrophy, and hepatocyte vacuolation. Both liver weight increases and liver histopathology were dose related and exposure duration dependent. A NOAEL of 10 mg/kg/day was selected based on the statistically significant incidence of hepatocytic hypertrophy at TCP doses ≥25 mg/kg/day. The NOAEL of 10 mg/kg/day is in agreement with the liver histopathology results reported by Hattula et al. [8], but below the NOAEL of 25 mg/kg/day selected by IRIS [18] based on increased liver weights and centrilobular hypertrophy at TCP doses ≥100 mg/kg/day.

## Supplementary Material

Supplemental Table 1: provides individual animal body weights and liver weights of male Sprague Dawley rats assigned to the TCP study. Assigned animal numbers and TCP dose groups are provided. Study time points of 5 days, 2 weeks, 4 weeks, and 13 weeks and dates of necropsy are provided. Calculations of individual animal relative liver weights *(*percentage of body weight) and TCP dose group means and standard deviations are also provided.

## Figures and Tables

**Table 1:** Study design showing number of male SD rats assigned per TCP dose group and time point.

Time point (animal necropsy)	TCP dose (mg/kg/day)^1^					
	0^2^	10	25	50	100	200
5 days	11	10	10	10	10	10
2 weeks	11	10	10	10	10	10
4 weeks	11	10	10	10	10	10
13 weeks	12	10	10	10	10	10

^1^TCP doses were prepared in olive oil vehicle and administered daily, 7 days/week.

^2^Olive oil only.

**Table 2:** Blood TCP concentration (*μ*g/mL) in male SD rats following 5 days, 2 weeks, 4 weeks, and 13 weeks of daily exposure of TCP by oral gavage.

Time point	TCP dose (mg/kg/day)					
	0	10	25	50	100
5 days	0.0 ± 0.0^1^	0.8 ± 0.1	2.8 ± 0.9	6.6 ± 2.7	9.5 ± 2.6	19.6 ± 5.7
2 weeks	0.0 ± 0.0	1.2 ± 0.4	6.7 ± 1.6	11.3 ± 5.4^2^	14.0 ± 1.7	16.7 ± 2.0
4 weeks	0.0 ± 0.0	0.8 ± 0.5	2.3 ± 1.0	5.6 ± 2.3	8.0 ± 2.7	15.4 ± 4.4
13 weeks	0.0 ± 0.0	1.3 ± 0.7	2.2 ± 0.7	5.3 ± 1.7	8.7 ± 2.9	8.5 ± 2.0

^1^Values are mean ± standard deviation (*n* = 5).

^2^ *n* = 6.

**Table 3:** Select serum chemistry concentrations (IU/L) of male SD rats following 5 days, 2 weeks, 4 weeks, and 13 weeks of daily exposure of TCP by oral gavage.

Time point	Analyte	TCP dose (mg/kg/day)					
		0	10	25	50	100	200
5 Days	ALP^1^	621 ± 204^4^	515 ± 205	681 ± 212	585 ± 163	610 ± 106	761 ± 178
	ALT^2^	40 ± 8	35 ± 10	38 ± 11	41 ± 12	41 ± 20	50 ± 15
	AST^3^	118 ± 14	109 ± 36	116 ± 36	118 ± 25	129 ± 33	126 ± 31

2 Weeks	ALP	511 ± 219	537 ± 140	555 ± 146	564 ± 209	566 ± 187	653 ± 120
	ALT	40 ± 13	40 ± 11	52 ± 51	31 ± 11	42 ± 13	68 ± 21**
	AST	126 ± 56	127 ± 42	137 ± 83	116 ± 51	132 ± 36	144 ± 18

4 Weeks	ALP	407 ± 128	500 ± 140	463 ± 118	489 ± 98	530 ± 180	523 ± 123
	ALT	43 ± 9	39 ± 8	39 ± 9	38 ± 8	47 ± 10	57 ± 10*
	AST	122 ± 23	134 ± 20	129 ± 25	119 ± 14	123 ± 11	124 ± 13

13 Weeks	ALP	304 ± 83	295 ± 102	396 ± 153	357 ± 115	425 ± 115	585 ± 127**
	ALT	38 ± 14	42 ± 9	57 ± 52	61 ± 39*	63 ± 29*	120 ± 47**
	AST	125 ± 24	130 ± 17	137 ± 21	152 ± 45	151 ± 22	244 ± 59**

^1^ALP: alkaline phosphatase.

^2^ALT: alanine aminotransferase.

^3^AST: aspartate aminotransferase.

^4^mean ± standard deviation for *n* = 10 except for the following groups: *n* = 9 (25 and 50 mg/kg/day, 13 weeks); *n* = 11 (0 mg/kg/day, 5 days, 2 weeks, and 4 weeks); *n* = 12 (0 mg/kg/day, 13 weeks).

*P < 0.05 compared to control.

**P < 0.01 compared to control.

**Table 4:** Absolute and relative (to body weight) liver weights of male SD rats following 5 days, 2 weeks, 4 weeks, and 13 weeks of daily exposure of TCP by oral gavage.

Time point	Liver weight	TCP dose (mg/kg/day)					
		0	10	25	50	100	200
5 Days	g^1^	13.1 ± 1.1^3^	13.4 ± 1.7	13.5 ± 1.3	13.1 ± 2.0	15.6 ± 1.7**	16.1 ± 1.4***
	%^2^	4.36 ± 0.20	4.44 ± 0.29	4.48 ± 0.35	4.58 ± 0.37	5.16 ± 0.67***	5.48 ± 0.32***
2 Weeks	g	13.8 ± 1.5	15.2 ± 1.3	15.9 ± 1.7*	17.9 ± 2.1***	19.5 ± 1.8***	21.4 ± 2.3***
	%	3.89 ± 0.24	4.25 ± 0.27*	4.43 ± 0.25***	4.96 ± 0.34***	5.56 ± 0.23***	6.36 ± 0.32***
4 Weeks	g	16.0 ± 3.3	16.4 ± 2.1	17.4 ± 2.6	20.0 ± 3.7*	23.2 ± 2.0***	25.4 ± 4.8***
	%	3.90 ± 0.68	3.86 ± 0.24	4.23 ± 0.28	4.73 ± 0.49**	5.75 ± 0.32***	6.54 ± 0.78***
13 Weeks	g	16.8 ± 2.9	21.4 ± 2.7	24.2 ± 3.3**	27.5 ± 5.5***	33.6 ± 7.3***	38.9 ± 7.2***
	%	3.10 ± 0.20	3.65 ± 0.13	4.36 ± 0.42***	5.46 ± 0.62***	7.11 ± 0.86***	9.40 ± 1.11***

^1 ^g: grams.

^2^%: [liver weight/body weight] × 100.

^3^mean ± standard deviation for *n* = 10 except for the following groups: *n* = 9 (25 mg/kg/day and 50 mg/kg/day, 13 weeks); *n* = 11 (0 mg/kg/day, 5 days, 2 weeks, and 4 weeks); *n* = 12 (0 mg/kg/day, 13 weeks).

*P < 0.05 compared to control.

**P < 0.01 compared to control.

***P < 0.001 compared to control.

**Table 5:** Selected histopathologic changes in the hepatocyte of male SD rats following 5 days, 2 weeks, 4 weeks, and 13 weeks of daily exposure of TCP by oral gavage.

Time point	Finding	TCP dose (mg/kg/day)					
		0	10	25	50	100	200
5 days	Vac-C^1^	—^6^	—	—	—	—	—
	Hyp-C^2^	—	—	—	—	1/10 (1.0)	10/10*** (1.0)
	Nec-C^3^	—	—	—	—	1/10 (1.0)	2/10 (1.0)

2 weeks	Vac-C	—	—	1/10 (1.0)^7^	1/10 (1.0)	4/10 (1.5)	7/10** (1.6)
	Hyp-C	—	—	—	4/10 (1.0)	10/10*** (2.0)	10/10*** (3.4)
	Nec-C	—	—	—	2/10 (1.0)	6/10* (1.0)	9/10*** (2.3)

4 weeks	Vac-C	—	1/10 (1.0)	4/10 (1.3)	7/10** (1.4)	9/10*** (1.7)	8/10** (1.6)
	Hyp-C	—	—	4/10 (1.0)	10/10*** (1.0)	10/10*** (2.2)	10/10*** (3.5)
	Nec-C	—	—	—	7/10** (1.0)	9/10*** (1.3)	9/10*** (2.4)

13 weeks	Vac-C	4/12 (1.0)	9/10 (1.6)	9/9* (2.4)	9/9* (3.4)	10/10** (4.3)	10/10** (4.7)
	Hyp-C	—	4/10 (1.0)	8/9*** (1.3)	9/9*** (2.6)	—	—
	Hyp-D^4^	—	—	—	—	10/10*** (3.0)	10/10*** (4.2)
	Nec-C	—	—	—	3/9 (1.0)	2/10 (1.5)	—
	Nec-M^5^	—	—	—	—	1/10 (1.0)	10/10*** (2.3)

^1^Vac-C: vacuolation (centrilobular).

^2^Hyp-C: hypertrophy (centrilobular).

^3^Nec-C: necrosis, single cell (centrilobular).

^4^Hyp-D: hypertrophy (diffuse).

^5^Nec-M: necrosis, single cell (midzonal).

^6^— indicates no finding.

^7^Incidence: number with finding/number examined (average severity score where 1: minimal, 2: slight/mild, 3: moderate, 4: moderately severe, 5: severe/high).

*P < 0.05 compared to control.

**P < 0.01 compared to control.

***P < 0.001 compared to control.
