# Supplementary material for: Subchronic Hepatotoxicity Evaluation of 2,3,4,6-Tetrachlorophenol in Sprague Dawley Rats
Source: J Toxicol. 2012 May 15;2012:376246. doi: 10.1155/2012/376246 (PMC3362204; doi:10.1155/2012/376246)
Supplement: Supplementary file 1 — Supplemental Table 1: provides individual animal body weights and liver weights of male Sprague Dawley rats assigned to the TCP study. Assigned animal numbers and TCP dose groups are provided. Study time points of 5 days, 2 weeks, 4 weeks, and 13 weeks and dates of necropsy are provided. Calculations of individual animal relative liver weights (percentage of body weight) and TCP dose group means and standard deviations are also provided. [file 376246.f1.pdf]

Supplemental Table 1

Individual Animal Data

5-Day

Animal #	Treatment	Dose	Necropsy Date	Body Weight	Liver Weight	Liver Weight as % of Body Weight
521	Olive Oil 1	0	8/4/2010	283.7	12.352	4.35
522	Olive Oil 1	0	8/4/2010	273.2	11.844	4.34
523	Olive Oil 1	0	8/4/2010	289.0	12.350	4.27
524	Olive Oil 1	0	8/4/2010	322.4	13.907	4.31
525	Olive Oil 1	0	8/4/2010	306.4	14.339	4.68
526	Olive Oil 1	0	8/4/2010	293.8	11.509	3.92
527	Olive Oil 1	0	8/4/2010	332.1	14.513	4.37
528	Olive Oil 1	0	8/4/2010	268.5	11.984	4.46
529	Olive Oil 1	0	8/4/2010	298.9	13.312	4.45
530	Olive Oil 1	0	8/4/2010	345.0	14.422	4.18
761	Olive Oil 1	0	8/4/2010	292.3	13.419	4.59
Mean				300.5	13.086	4.36
Standard Deviation				24.1	1.120	0.20
531	2,3,4,6-Tetrachlorophenol	10	8/4/2010	256.7	11.175	4.35
532	2,3,4,6-Tetrachlorophenol	10	8/4/2010	321.9	16.199	5.03
533	2,3,4,6-Tetrachlorophenol	10	8/4/2010	302.1	13.293	4.40

534	2,3,4,6-Tetrachlorophenol	10	8/4/2010	267.5	11.812	4.42
535	2,3,4,6-Tetrachlorophenol	10	8/4/2010	296.7	12.236	4.12
536	2,3,4,6-Tetrachlorophenol	10	8/4/2010	306.4	13.942	4.55
537	2,3,4,6-Tetrachlorophenol	10	8/4/2010	335.7	15.317	4.56
538	2,3,4,6-Tetrachlorophenol	10	8/4/2010	330.8	14.508	4.39
539	2,3,4,6-Tetrachlorophenol	10	8/4/2010	284.2	11.241	3.96
540	2,3,4,6-Tetrachlorophenol	10	8/4/2010	306.0	14.282	4.67
Mean				300.8	13.401	4.44
Standard Deviation				25.8	1.742	0.29
541	2,3,4,6-Tetrachlorophenol	25	8/4/2010	291.0	14.826	5.09
542	2,3,4,6-Tetrachlorophenol	25	8/4/2010	306.8	15.465	5.04
543	2,3,4,6-Tetrachlorophenol	25	8/4/2010	306.2	14.264	4.66
544	2,3,4,6-Tetrachlorophenol	25	8/4/2010	330.7	14.303	4.33
545	2,3,4,6-Tetrachlorophenol	25	8/4/2010	317.7	14.013	4.41
546	2,3,4,6-Tetrachlorophenol	25	8/4/2010	302.2	13.052	4.32
547	2,3,4,6-Tetrachlorophenol	25	8/4/2010	278.0	11.330	4.08
548	2,3,4,6-Tetrachlorophenol	25	8/4/2010	309.9	13.189	4.26
549	2,3,4,6-Tetrachlorophenol	25	8/4/2010	281.5	11.656	4.14
550	2,3,4,6-Tetrachlorophenol	25	8/4/2010	297.5	13.228	4.45
Mean				302.2	13.533	4.48
Standard Deviation				16.0	1.316	0.35
551	2,3,4,6-Tetrachlorophenol	50	8/4/2010	233.3	11.060	4.74
552	2,3,4,6-Tetrachlorophenol	50	8/4/2010	307.6	16.236	5.28
553	2,3,4,6-Tetrachlorophenol	50	8/4/2010	236.9	10.882	4.59
554	2,3,4,6-Tetrachlorophenol	50	8/4/2010	313.9	15.337	4.89

555	2,3,4,6-Tetrachlorophenol	50	8/4/2010	319.9	14.133	4.42
556	2,3,4,6-Tetrachlorophenol	50	8/4/2010	284.6	13.079	4.60
557	2,3,4,6-Tetrachlorophenol	50	8/4/2010	301.5	14.069	4.67
558	2,3,4,6-Tetrachlorophenol	50	8/4/2010	297.0	12.641	4.26
559	2,3,4,6-Tetrachlorophenol	50	8/4/2010	296.5	13.185	4.45
560	2,3,4,6-Tetrachlorophenol	50	8/4/2010	256.0	10.003	3.91
Mean				284.7	13.063	4.58
Standard Deviation				31.5	1.992	0.37
561	2,3,4,6-Tetrachlorophenol	100	8/4/2010	256.0	17.138	6.69
562	2,3,4,6-Tetrachlorophenol	100	8/4/2010	326.1	14.612	4.48
563	2,3,4,6-Tetrachlorophenol	100	8/4/2010	292.5	15.965	5.46
564	2,3,4,6-Tetrachlorophenol	100	8/4/2010	343.5	18.772	5.46
565	2,3,4,6-Tetrachlorophenol	100	8/4/2010	300.6	13.726	4.57
566	2,3,4,6-Tetrachlorophenol	100	8/4/2010	291.9	13.309	4.56
567	2,3,4,6-Tetrachlorophenol	100	8/4/2010	295.1	14.857	5.03
568	2,3,4,6-Tetrachlorophenol	100	8/4/2010	260.7	14.429	5.53
569	2,3,4,6-Tetrachlorophenol	100	8/4/2010	344.2	16.552	4.81
570	2,3,4,6-Tetrachlorophenol	100	8/4/2010	325.7	16.291	5.00
Mean				303.6	15.565	5.16
Standard Deviation				31.1	1.686	0.67
571	2,3,4,6-Tetrachlorophenol	200	8/4/2010	262.8	15.245	5.80
572	2,3,4,6-Tetrachlorophenol	200	8/4/2010	297.4	16.673	5.61
573	2,3,4,6-Tetrachlorophenol	200	8/4/2010	293.4	15.570	5.31
574	2,3,4,6-Tetrachlorophenol	200	8/4/2010	327.6	17.221	5.26
575	2,3,4,6-Tetrachlorophenol	200	8/4/2010	297.6	14.752	4.96

576	2,3,4,6-Tetrachlorophenol	200	8/4/2010	308.7	16.534	5.36
577	2,3,4,6-Tetrachlorophenol	200	8/4/2010	309.1	18.664	6.04
578	2,3,4,6-Tetrachlorophenol	200	8/4/2010	291.9	16.541	5.67
579	2,3,4,6-Tetrachlorophenol	200	8/4/2010	261.8	13.663	5.22
580	2,3,4,6-Tetrachlorophenol	200	8/4/2010	288.5	16.210	5.62
Mean				293.9	16.107	5.48
Standard Deviation				20.1	1.387	0.32

2-Week

Animal #	Treatment	Dose	Necropsy Date	Body Weight	Liver Weight	Liver Weight as % of Body Weight
581	Olive Oil 1	0	8/13/2010	317.8	11.009	3.46
582	Olive Oil 1	0	8/13/2010	341.9	14.147	4.14
583	Olive Oil 1	0	8/13/2010	348.8	14.421	4.13
584	Olive Oil 1	0	8/13/2010	342.7	12.442	3.63
585	Olive Oil 1	0	8/13/2010	365.1	13.976	3.83
586	Olive Oil 1	0	8/13/2010	363.9	14.069	3.87
587	Olive Oil 1	0	8/13/2010	372.2	15.298	4.11
588	Olive Oil 1	0	8/13/2010	325.8	12.400	3.81
589	Olive Oil 1	0	8/13/2010	394.8	16.633	4.21
590	Olive Oil 1	0	8/13/2010	371.2	13.963	3.76
762	Olive Oil 1	0	8/13/2010	355.2	13.587	3.83
Mean				354.5	13.813	3.89
Standard Deviation				22.2	1.501	0.24

591	2,3,4,6-Tetrachlorophenol	10	8/13/2010	316.0	13.178	4.17
592	2,3,4,6-Tetrachlorophenol	10	8/13/2010	367.0	15.738	4.29
593	2,3,4,6-Tetrachlorophenol	10	8/13/2010	382.5	15.865	4.15
594	2,3,4,6-Tetrachlorophenol	10	8/13/2010	331.6	12.809	3.86
595	2,3,4,6-Tetrachlorophenol	10	8/13/2010	398.5	16.174	4.06
596	2,3,4,6-Tetrachlorophenol	10	8/13/2010	340.3	16.556	4.87
597	2,3,4,6-Tetrachlorophenol	10	8/13/2010	333.4	14.134	4.24
598	2,3,4,6-Tetrachlorophenol	10	8/13/2010	385.1	16.056	4.17
599	2,3,4,6-Tetrachlorophenol	10	8/13/2010	365.9	15.249	4.17
600	2,3,4,6-Tetrachlorophenol	10	8/13/2010	350.2	15.782	4.51
Mean				357.1	15.154	4.25
Standard Deviation				27.0	1.313	0.27

601	2,3,4,6-Tetrachlorophenol	25	8/13/2010	383.6	18.287	4.77
602	2,3,4,6-Tetrachlorophenol	25	8/13/2010	357.4	15.628	4.37
603	2,3,4,6-Tetrachlorophenol	25	8/13/2010	333.6	15.274	4.58
604	2,3,4,6-Tetrachlorophenol	25	8/13/2010	341.6	14.340	4.20
605	2,3,4,6-Tetrachlorophenol	25	8/13/2010	361.8	16.704	4.62
606	2,3,4,6-Tetrachlorophenol	25	8/13/2010	351.0	14.876	4.24
607	2,3,4,6-Tetrachlorophenol	25	8/13/2010	361.8	17.006	4.70
608	2,3,4,6-Tetrachlorophenol	25	8/13/2010	373.9	17.000	4.55
609	2,3,4,6-Tetrachlorophenol	25	8/13/2010	313.0	12.742	4.07
610	2,3,4,6-Tetrachlorophenol	25	8/13/2010	412.8	17.244	4.18
Mean				359.1	15.910	4.43
Standard Deviation				27.6	1.650	0.25

611	2,3,4,6-Tetrachlorophenol	50	8/13/2010	364.1	18.057	4.96
612	2,3,4,6-Tetrachlorophenol	50	8/13/2010	367.7	18.165	4.94
613	2,3,4,6-Tetrachlorophenol	50	8/13/2010	352.7	14.885	4.22
614	2,3,4,6-Tetrachlorophenol	50	8/13/2010	398.1	19.265	4.84
615	2,3,4,6-Tetrachlorophenol	50	8/13/2010	391.2	21.272	5.44
616	2,3,4,6-Tetrachlorophenol	50	8/13/2010	374.6	18.469	4.93
617	2,3,4,6-Tetrachlorophenol	50	8/13/2010	376.2	18.244	4.85
618	2,3,4,6-Tetrachlorophenol	50	8/13/2010	361.8	19.278	5.33
619	2,3,4,6-Tetrachlorophenol	50	8/13/2010	314.6	16.543	5.26
620	2,3,4,6-Tetrachlorophenol	50	8/13/2010	301.4	14.459	4.80
Mean				360.2	17.864	4.96
Standard Deviation				30.8	2.066	0.34

621	2,3,4,6-Tetrachlorophenol	100	8/13/2010	330.7	16.838	5.09
622	2,3,4,6-Tetrachlorophenol	100	8/13/2010	366.5	21.288	5.81
623	2,3,4,6-Tetrachlorophenol	100	8/13/2010	323.5	18.633	5.76
624	2,3,4,6-Tetrachlorophenol	100	8/13/2010	328.5	18.787	5.72
625	2,3,4,6-Tetrachlorophenol	100	8/13/2010	372.5	21.066	5.66
626	2,3,4,6-Tetrachlorophenol	100	8/13/2010	382.6	20.641	5.39
627	2,3,4,6-Tetrachlorophenol	100	8/13/2010	359.2	20.391	5.68
628	2,3,4,6-Tetrachlorophenol	100	8/13/2010	304.8	16.284	5.34
629	2,3,4,6-Tetrachlorophenol	100	8/13/2010	364.4	20.707	5.68
630	2,3,4,6-Tetrachlorophenol	100	8/13/2010	380.8	20.836	5.47
Mean				351.4	19.547	5.56
Standard Deviation				27.2	1.815	0.23

631	2,3,4,6-Tetrachlorophenol	200	8/13/2010	348.2	22.730	6.53
632	2,3,4,6-Tetrachlorophenol	200	8/13/2010	344.1	21.601	6.28
633	2,3,4,6-Tetrachlorophenol	200	8/13/2010	335.2	19.485	5.81
634	2,3,4,6-Tetrachlorophenol	200	8/13/2010	347.2	21.590	6.22
635	2,3,4,6-Tetrachlorophenol	200	8/13/2010	351.8	24.204	6.88
636	2,3,4,6-Tetrachlorophenol	200	8/13/2010	277.3	17.425	6.28
637	2,3,4,6-Tetrachlorophenol	200	8/13/2010	363.5	22.212	6.11
638	2,3,4,6-Tetrachlorophenol	200	8/13/2010	358.4	23.350	6.52
639	2,3,4,6-Tetrachlorophenol	200	8/13/2010	293.6	18.282	6.23
640	2,3,4,6-Tetrachlorophenol	200	8/13/2010	345.7	23.421	6.77
Mean				336.5	21.430	6.36
Standard Deviation				28.3	2.296	0.32

4-Week

Animal #	Treatment	Dose	Necropsy Date	Body Weight	Liver Weight	Liver Weight as % of Body Weight
641	Olive Oil 1	0	8/27/2010	481.4	20.390	4.24
642	Olive Oil 1	0	8/27/2010	468.0	20.240	4.32
643	Olive Oil 1	0	8/27/2010	377.6	12.740	3.37
644	Olive Oil 1	0	8/27/2010	372.8	21.040	5.64
645	Olive Oil 1	0	8/27/2010	433.8	17.760	4.09
646	Olive Oil 1	0	8/27/2010	389.8	14.350	3.68
647	Olive Oil 1	0	8/27/2010	392.5	13.490	3.44
648	Olive Oil 1	0	8/27/2010	424.7	15.990	3.77
649	Olive Oil 1	0	8/27/2010	377.7	12.540	3.32
650	Olive Oil 1	0	8/27/2010	399.6	14.340	3.59
763	Olive Oil 1	0	8/27/2010	378.1	12.950	3.43
Mean				408.7	15.985	3.90
Standard				38.1	3.308	0.68

Deviation						
651	2,3,4,6-Tetrachlorophenol	10	8/27/2010	438.3	18.590	4.24
652	2,3,4,6-Tetrachlorophenol	10	8/27/2010	405.3	16.510	4.07
653	2,3,4,6-Tetrachlorophenol	10	8/27/2010	439.3	15.390	3.50
654	2,3,4,6-Tetrachlorophenol	10	8/27/2010	397.3	16.290	4.10
655	2,3,4,6-Tetrachlorophenol	10	8/27/2010	485.2	18.750	3.86
656	2,3,4,6-Tetrachlorophenol	10	8/27/2010	337.0	12.480	3.70
657	2,3,4,6-Tetrachlorophenol	10	8/27/2010	429.1	16.210	3.78
658	2,3,4,6-Tetrachlorophenol	10	8/27/2010	411.4	14.950	3.63
659	2,3,4,6-Tetrachlorophenol	10	8/27/2010	415.1	15.460	3.72
660	2,3,4,6-Tetrachlorophenol	10	8/27/2010	485.6	19.450	4.01
Mean				424.4	16.408	3.86
Standard Deviation				43.3	2.083	0.24
661	2,3,4,6-Tetrachlorophenol	25	8/27/2010	404.0	17.870	4.42
662	2,3,4,6-Tetrachlorophenol	25	8/27/2010	497.9	22.490	4.52
663	2,3,4,6-Tetrachlorophenol	25	8/27/2010	444.5	17.480	3.93
664	2,3,4,6-Tetrachlorophenol	25	8/27/2010	357.3	15.420	4.32
665	2,3,4,6-Tetrachlorophenol	25	8/27/2010	457.9	21.070	4.60
666	2,3,4,6-Tetrachlorophenol	25	8/27/2010	406.6	18.320	4.51
667	2,3,4,6-Tetrachlorophenol	25	8/27/2010	397.4	15.350	3.86
668	2,3,4,6-Tetrachlorophenol	25	8/27/2010	384.1	14.810	3.86
669	2,3,4,6-Tetrachlorophenol	25	8/27/2010	360.6	15.180	4.21
670	2,3,4,6-Tetrachlorophenol	25	8/27/2010	400.2	16.480	4.12
Mean				411.1	17.447	4.23
Standard				44.0	2.604	0.28

Deviation						
671	2,3,4,6-Tetrachlorophenol	50	8/27/2010	461.4	24.330	5.27
672	2,3,4,6-Tetrachlorophenol	50	8/27/2010	415.7	21.170	5.09
673	2,3,4,6-Tetrachlorophenol	50	8/27/2010	415.9	18.500	4.45
674	2,3,4,6-Tetrachlorophenol	50	8/27/2010	484.2	25.950	5.36
675	2,3,4,6-Tetrachlorophenol	50	8/27/2010	443.7	20.770	4.68
676	2,3,4,6-Tetrachlorophenol	50	8/27/2010	375.3	14.130	3.76
677	2,3,4,6-Tetrachlorophenol	50	8/27/2010	388.0	17.280	4.45
678	2,3,4,6-Tetrachlorophenol	50	8/27/2010	406.7	19.910	4.90
679	2,3,4,6-Tetrachlorophenol	50	8/27/2010	360.2	15.680	4.35
680	2,3,4,6-Tetrachlorophenol	50	8/27/2010	445.9	21.990	4.93
Mean				419.7	19.971	4.73
Standard Deviation				39.3	3.694	0.49
681	2,3,4,6-Tetrachlorophenol	100	8/27/2010	400.3	22.630	5.65
682	2,3,4,6-Tetrachlorophenol	100	8/27/2010	404.2	21.650	5.36
683	2,3,4,6-Tetrachlorophenol	100	8/27/2010	416.6	24.890	5.97
684	2,3,4,6-Tetrachlorophenol	100	8/27/2010	347.9	19.740	5.67
685	2,3,4,6-Tetrachlorophenol	100	8/27/2010	408.5	26.280	6.43
686	2,3,4,6-Tetrachlorophenol	100	8/27/2010	396.0	21.660	5.47
687	2,3,4,6-Tetrachlorophenol	100	8/27/2010	413.0	25.010	6.06
688	2,3,4,6-Tetrachlorophenol	100	8/27/2010	445.6	24.470	5.49
689	2,3,4,6-Tetrachlorophenol	100	8/27/2010	417.8	24.030	5.75
690	2,3,4,6-Tetrachlorophenol	100	8/27/2010	383.6	21.680	5.65
Mean				403.4	23.204	5.75
Standard				25.4	2.036	0.32

Deviation						
691	2,3,4,6-Tetrachlorophenol	200	8/27/2010	373.7	21.710	5.81
692	2,3,4,6-Tetrachlorophenol	200	8/27/2010	447.7	38.070	8.50
693	2,3,4,6-Tetrachlorophenol	200	8/27/2010	382.1	24.080	6.30
694	2,3,4,6-Tetrachlorophenol	200	8/27/2010	368.4	23.430	6.36
695	2,3,4,6-Tetrachlorophenol	200	8/27/2010	346.1	24.170	6.98
696	2,3,4,6-Tetrachlorophenol	200	8/27/2010	352.1	21.100	5.99
697	2,3,4,6-Tetrachlorophenol	200	8/27/2010	424.0	25.310	5.97
698	2,3,4,6-Tetrachlorophenol	200	8/27/2010	394.5	24.360	6.17
699	2,3,4,6-Tetrachlorophenol	200	8/27/2010	407.7	27.380	6.72
700	2,3,4,6-Tetrachlorophenol	200	8/27/2010	366.1	24.070	6.57
Mean				386.2	25.368	6.54
Standard Deviation				32.3	4.790	0.78

13-Week

Animal #	Treatment	Dose	Necropsy Date	Body Weight	Liver Weight	Liver Weight as % of Body Weight
701	Olive Oil 1	0	10/28/2010	533.9	16.802	3.15
702	Olive Oil 1	0	10/28/2010	735.4	23.956	3.26
703	Olive Oil 1	0	10/28/2010	596.4	16.372	2.75
704	Olive Oil 1	0	10/28/2010	555.2	17.990	3.24
705	Olive Oil 1	0	10/28/2010	518.8	17.537	3.38
706	Olive Oil 1	0	10/28/2010	485.5	13.946	2.87
707	Olive Oil 1	0	10/28/2010	451.3	13.834	3.07
708	Olive Oil 1	0	10/28/2010	519.8	15.109	2.91

709	Olive Oil 1	0	10/28/2010	603.3	19.781	3.28
710	Olive Oil 1	0	10/28/2010	513.1	16.943	3.30
764	Olive Oil 1	0	10/28/2010	499.3	14.824	2.97
765	Olive Oil 1	0	10/28/2010	466.2	14.190	3.04
Mean				539.9	16.774	3.10
Standard Deviation				77.0	2.904	0.20
711	2,3,4,6-Tetrachlorophenol	10	10/28/2010	644.6	22.706	3.52
712	2,3,4,6-Tetrachlorophenol	10	10/28/2010	633.9	23.709	3.74
713	2,3,4,6-Tetrachlorophenol	10	10/28/2010	576.9	21.340	3.70
714	2,3,4,6-Tetrachlorophenol	10	10/28/2010	660.0	25.781	3.91
715	2,3,4,6-Tetrachlorophenol	10	10/28/2010	528.5	19.356	3.66
716	2,3,4,6-Tetrachlorophenol	10	10/28/2010	636.9	23.866	3.75
717	2,3,4,6-Tetrachlorophenol	10	10/28/2010	531.0	18.259	3.44
718	2,3,4,6-Tetrachlorophenol	10	10/28/2010	511.5	18.181	3.55
719	2,3,4,6-Tetrachlorophenol	10	10/28/2010	609.8	22.332	3.66
720	2,3,4,6-Tetrachlorophenol	10	10/28/2010	509.1	18.298	3.59
Mean				584.2	21.383	3.65
Standard Deviation				59.8	2.733	0.13
721	2,3,4,6-Tetrachlorophenol	25	10/28/2010	549.1	27.986	5.10
722	2,3,4,6-Tetrachlorophenol	25	10/28/2010	589.9	26.622	4.51
723	2,3,4,6-Tetrachlorophenol	25	10/28/2010	482.4	19.680	4.08
724	2,3,4,6-Tetrachlorophenol	25	10/28/2010	507.0	23.214	4.58
725	2,3,4,6-Tetrachlorophenol	25	10/28/2010	527.4	21.897	4.15
726	2,3,4,6-Tetrachlorophenol	25	10/28/2010	659.6	27.901	4.23
727	2,3,4,6-Tetrachlorophenol	25	10/28/2010	500.3	20.714	4.14
729	2,3,4,6-Tetrachlorophenol	25	10/28/2010	577.7	27.597	4.78

730	2,3,4,6-Tetrachlorophenol	25	10/28/2010	593.6	22.002	3.71
Mean				554.1	24.179	4.36
Standard Deviation				56.5	3.338	0.42
731	2,3,4,6-Tetrachlorophenol	50	10/28/2010	391.8	17.996	4.59
732	2,3,4,6-Tetrachlorophenol	50	10/28/2010	516.6	28.270	5.47
734	2,3,4,6-Tetrachlorophenol	50	10/28/2010	496.4	27.140	5.47
735	2,3,4,6-Tetrachlorophenol	50	10/28/2010	484.4	28.113	5.80
736	2,3,4,6-Tetrachlorophenol	50	10/28/2010	471.0	25.167	5.34
737	2,3,4,6-Tetrachlorophenol	50	10/28/2010	502.0	24.588	4.90
738	2,3,4,6-Tetrachlorophenol	50	10/28/2010	546.7	30.602	5.60
739	2,3,4,6-Tetrachlorophenol	50	10/28/2010	521.0	26.899	5.16
740	2,3,4,6-Tetrachlorophenol	50	10/28/2010	571.2	38.757	6.79
Mean				500.1	27.504	5.46
Standard Deviation				51.0	5.501	0.62
741	2,3,4,6-Tetrachlorophenol	100	10/28/2010	467.3	32.886	7.04
742	2,3,4,6-Tetrachlorophenol	100	10/28/2010	332.6	18.134	5.45
743	2,3,4,6-Tetrachlorophenol	100	10/28/2010	567.1	45.946	8.10
744	2,3,4,6-Tetrachlorophenol	100	10/28/2010	424.1	34.486	8.13
745	2,3,4,6-Tetrachlorophenol	100	10/28/2010	487.4	32.085	6.58
746	2,3,4,6-Tetrachlorophenol	100	10/28/2010	490.0	34.275	6.99
747	2,3,4,6-Tetrachlorophenol	100	10/28/2010	499.2	34.699	6.95
748	2,3,4,6-Tetrachlorophenol	100	10/28/2010	434.0	27.808	6.41
749	2,3,4,6-Tetrachlorophenol	100	10/28/2010	493.4	39.823	8.07
750	2,3,4,6-Tetrachlorophenol	100	10/28/2010	487.8	36.045	7.39
Mean				468.3	33.619	7.11
Standard Deviation				61.6	7.266	0.86

751	2,3,4,6-Tetrachlorophenol	200	10/28/2010	394.6	40.915	10.37
752	2,3,4,6-Tetrachlorophenol	200	10/28/2010	427.4	36.220	8.47
753	2,3,4,6-Tetrachlorophenol	200	10/28/2010	446.2	36.599	8.20
754	2,3,4,6-Tetrachlorophenol	200	10/28/2010	403.5	44.742	11.09
755	2,3,4,6-Tetrachlorophenol	200	10/28/2010	341.1	31.095	9.12
756	2,3,4,6-Tetrachlorophenol	200	10/28/2010	415.5	38.831	9.35
757	2,3,4,6-Tetrachlorophenol	200	10/28/2010	429.9	36.627	8.52
758	2,3,4,6-Tetrachlorophenol	200	10/28/2010	348.7	28.621	8.21
759	2,3,4,6-Tetrachlorophenol	200	10/28/2010	491.5	54.316	11.05
760	2,3,4,6-Tetrachlorophenol	200	10/28/2010	428.1	41.387	9.67
Mean				412.7	38.935	9.40
Standard Deviation				44.4	7.198	1.11
